# Department of Health

**Published:** 1908-11

**Authors:** Ernest Wende

**Affiliations:** Health Commissioner. Municipal Building, Buffalo


### DEPARTMENT OF HEALTH.

Municipal Building,
Buffalo, October 12, 1908.

*To the Committee of Contagious Disease Hospital, Buffalo Academy of Medicine:*

Gentlemen: In considering the existing necessity for contagious disease hospital, the fact is referred to that since the organisation of the Department of Health, the establishment of such institution has been part of the official program, its steadily increasing want emphasised and provisions for its establishment continuously recommended.

Throughout Dr. Greene's administration, it was likewise continuously advocated and has continued to be so up to the present time. Failure to secure provision for it has been a constant serious drawback and has deprived the municipality of the most important factor in contending with and diminishing contagion; for the contagious sick, which should now receive hospital care, have of necessity to be left scattered throughout a populous community under conditions where protection is difficult, and dissemination, to say nothing of the humanitarian side of the question. It is only necessary to look over the field to which it would administer, to emphasise the urgent want.

Among the poorer classes, a visitation of contagious sickness means unexpected, unprepared-for expense—often causes concealment with resulting certain spread of disease, for publicity too frequently means loss of work and finally increases the demands on the Poor Department.

Buffalo, in its rapidly growing state, has a large transient population, residing in all grades of rooming houses, minor boarding places and where not. A visitation of contagious diseases among this class means hardship and suffering, economic loss, and, again, often concealment and spread of contagion, and the like.

During the year, thousands of strangers and visitors, are with us. They come from no class. Sickness is no discriminator. Whether in hotel or private house, the problem is again presented, and it is often from the stranger with us that the just criticism is made that, in a city of almost half a million, there is lack of provision for the contagious sick, and in this the municipality is morally and otherwise responsible. Irrespective of the environment or status of these cases, money, loss of work and hardship are accompaniments. Suffering is greater, mortality is higher, concealment frequent, and spread of contagion more or less inevitable.

Buffalo, has for years, enjoyed particular prestige in being foremost and aggressive in all measures for the Public Health—as evidenced, notably, in its public baths, laboratory work, food and milk surveillance, special investigations, rapid service, procedure and the like. The lack, however, of the most important factor in combating contagion—and hospital for their care and segregation—has brought it to a standstill, invited just criticism, that this city, with its large population, is behind the times, behind lesser municipalities in an equipment, the necessity for which permits no discussion; which is essential and which the want of, directly and indirectly, is the cause of suffering, sickness, death, want and economic loss. This reference would be incomplete were not the indication equally strong, the data and possibilities the same, the lack of means to combat the infection and care for the sick the same, in tuberculosis.

At no time has the public throughout the country been so well informed, interested and exacting as to means for its protection from and for the durability of the scourge of the race as now. Never has scientific work been more available in obtaining practical results, never activity more aggressive and expenditure more liberal, and durability and disseminations demonstrable. Of the many phases of the problem its treatment and cure is uppermost in the field of endeavor by nation, state and cities, and provision for exterminating this White Plague are established and being established on all sides. The position of the consumptive in our midst is most pitiable. With a knowledge that curability is a fact in its incipiency, there is no provision that he can readily avail himself of, and hope is darkened.

With the advanced case it is yet more sad. Being undesirable cases, hospitals are loath to receive them, for they are obliged to mingle with other sick, with all that it implies. So that from inadequate provision, the curable cases cannot get the facilities, while the incurable, pitiable as it is, unwelcome in our hospitals, and too frequently unwelcome at home, words cannot portray the mental despair and anguish in both classes of cases. The relation of general hospitals to this disease is unfortunate. Being unfitted to receive and treat them, they should not be admitted, for their admission means mingling with other patients with all that it implies—notwithstanding which, and that they are undesirable cases, they are perforce obliged, on occasions to accept them, though with results that are unfortunate, as has been recently demonstrated in our own city. In considering the question of hospital equipment, it is again urged that provision for tubercular patients be included. It is recommended that, for the curable incipient ones, hospital facilities with out-door treatment on the now accepted principles, be provided beyond the city limits; while, for the incurables, those who are and should be debarred from general hospitals, and who are not desired in the home circle, and who are constant sources of infection in our midst, a specially adapted institution should be organised. Until we do this, we must continue to pay the penalty, be the subject of just criticism, be behind other municipalities and be conscious of not doing our duty to the public and the sick.

In closing, no time need be occupied with the committee or profession in referring to the fact that while we have a smallpox hospital it in no way has any utilitarian bearing on the situation. Its distinctive character for strict isolation and treatment of pestilential disease, with which no other sickness can be even remotely assembled, are sufficient.

The recommendation, therefore, for a contagious hospital includes also that for the tubercular.

It was fully my intention to have been present at the meeting, but indisposition today, I regret to say, compels me to deny myself that privilege.

Most sincerely yours,

ERNEST WENDE,

Health Commissioner.

